# The Association of Olfactory Function with BMI, Appetite, and Prospective Weight Change in Dutch Community-Dwelling Older Adults

**DOI:** 10.1007/s12603-019-1241-7

**Published:** 2019-08-30

**Authors:** K.S. Fluitman, H.J. Nadar, D.S. Roos, H.W. Berendse, B.J.F. Keijser, M. Nieuwdorp, R.G. Ijzerman, M. Visser

**Affiliations:** 1 Department of Internal Medicine, Amsterdam UMC, Vrije Universiteit Amsterdam, Amsterdam Public Health Research Institute, De Boelelaan 1117, 1081 HV, Amsterdam, The Netherlands; 2 Wallenburg Laboratory, Department of Molecular and Clinical Medicine, Sahlgrenska Academy, University of Gothenburg, Gothenburg, Sweden; 3 Department of Health Sciences, Faculty of Science, Vrije Universiteit Amsterdam, Amsterdam Public Health Research Institute, Amsterdam, The Netherlands; 4 Department of Neurology, Amsterdam UMC, Vrije Universiteit Amsterdam, Amsterdam, The Netherlands; 5 Department of Microbiology and Systems biology, TNO earth, Life and Social Sciences, Zeist, The Netherlands; 6 Department of Preventive Dentistry, Academic Center for Dentistry Amsterdam, University of Amsterdam and Vrije Universiteit Amsterdam, Amsterdam, The Netherlands; 7 Department of Vascular Medicine, Amsterdam UMC, University of Amsterdam, Amsterdam, The Netherlands

**Keywords:** Olfactory function, BMI, appetite, weight, older adults

## Abstract

**Objectives:**

The olfactory decline that often accompanies aging is thought to contribute to undernutrition in older adults. It is believed to negatively affect eating pleasure, appetite, food intake and subsequently nutritional status. We have evaluated the associations of olfactory function with BMI, appetite and prospective weight change in a cohort of Dutch community-dwelling older adults.

**Design:**

Cross-sectional cohort study.

**Participants:**

Dutch community-dwelling older adults from the ongoing Longitudinal Aging Study Amsterdam (LASA).

**Measurements and setting:**

In 2012–2013, the 40-item University of Pennsylvania Smell Identification Test (UPSIT) was administered to 824 LASA participants to evaluate their olfactory function. Body weight, height, appetite, comorbidity, cognitive status and socio-demographic factors were also assessed. Follow-up weight was measured after three years.

**Results:**

673 participants (aged 55–65 years) were included in the regression analyses. Median UPSIT-score was 33. When adjusted for potential confounders, lower UPSIT-score (indicative of poorer olfactory function) was not associated with poor appetite (OR = 1.062, p = 0.137) or prospective weight change (B = −0.027, p = 0.548). It was, however, associated with lower BMI in smokers (B = 0.178, p = 0.032), but not in non-smokers (B = −0.015, p = 0.732).

**Conclusion:**

Lower olfactory function scores were associated with lower BMI in community-dwelling older adults who smoke, but not with appetite or prospective weight change. Therefore, smoking older adults with olfactory impairments may pose as a vulnerable group with respect to developing undernutrition.

## Introduction

An important health concern in the older population is protein-energy undernutrition (1). This is a form of malnutrition frequently resulting from insufficient energy or protein uptake (2). Its prevalence rates vary from 7–16% in community-dwelling older adults to 18–33% in institutionalized older adults (3). However, since about 95% of the Dutch older population lives at home, this is where most cases reside (4).

In order to develop effective preventive interventions, a wide interest in the determinants of undernutrition in older adults has emerged (5). The age-related decline in olfactory function (presbyosmia) is argued to be one of these determinants (6, 7). Presbyosmia affects up to 60% of the aged population (8, 9, 10, 11). It is hypothesized to cause appetite suppression and reduce food intake, increasing the risk of developing undernutrition (12, 13). Nevertheless, the potential importance of olfaction in relation to healthy ageing is underappreciated in comparison with other senses, such as hearing and vision (14). Also, current literature on the relationship between olfaction and nutritional status is inconsistent (11, 14, 15, 16, 17, 18, 19, 20). In a large cohort of 1636 older adults aged over 60, poorer olfactory function was indeed associated with lower BMI (20). In a sub-sample of 557 older adults from this cohort, Gopinath et al. found that women with olfactory impairment at baseline had lower dietary quality 5 years later (14). In addition, Seubert et al. studied 2234 older adults and also found olfactory dysfunction to be associated with both a BMI<18 and poor appetite (11). Conversely, it has been reported that olfactory dysfunction was not at all related to diminished eating pleasure or poor appetite (17) or to risk of malnutrition, measured by either low BMI or low Mini-Nutritional Assessment score (16, 18, 19). Aside from the study by Gopinath et al. (14) these studies were all cross-sectional (15, 16, 17, 18, 19, 20).

In the current study, we aimed to evaluate the relationship between impaired olfactory function (measured with an extensive 40-item smell identification test), poor appetite and low BMI in a large cohort of 824 community-dwelling Dutch older adults. In addition, we tested whether impaired olfactory function is associated with 3-year prospective weight change. If olfactory dysfunction indeed contributes to poor appetite and the development of undernutrition, dietary interventions should specifically be tailored to those older adults with an impaired sense of smell (12).

## Materials and Methods

### Subjects

Data were used from the ongoing, large, multidisciplinary Longitudinal Aging Study Amsterdam (LASA) (21). The main objective of LASA is to examine the predictors and consequences of ageing in community-dwelling Dutch older adults. This is described more elaborately elsewhere (21, 22).

In 2012–2013, a third LASA-cohort aged 55–65 years was included (n=1023). These 1023 participants were asked to undergo an additional olfactory test. Of them, 199 declined and 824 consented. The study was approved by the local medical ethics review board.

### Olfactory function

Olfactory function was measured by the self-administrated University of Pennsylvania Smell Identification Test (UPSIT) (23). The UPSIT-test consists of 40 different microencapsulated odors, which are released by scratching the microcapsule. Participants are then required to identify each odor by choosing from four forced-choice response options. Participants were also asked if they experienced any smell problems in general, with answering categories ‘yes' and ‘no'. Since the UPSIT-score is gender dependent, the following olfactory function categories were used as provided by the test manufacturer: normosmic (34–40 for male and 35–40 for female), microsmic (19–33 for male and 19–34 for female) and anosmic (<19 for both male and female) (24).

### Anthropometry

Body weight (kg) and height (m) were measured during the LASA interviews by trained interviewers. Measured weight was adjusted by subtracting 1 kg if the participant was wearing shoes, clothes, or a corset. In case of unavailable measurements, self-reported body weight or height were used. BMI (kg/m2) was calculated. Weight change was assessed by subtracting baseline weight from follow-up weight measurement in 2015–2016 (3-year follow-up).

### Appetite

Information on appetite was obtained using the second question from the Dutch translation of the Centre for Epidemiologic Studies Depression scale (CES-D) (25): “In the past week, I did not feel like eating, my appetite was poor”. Possible answers were: 1 “rarely or never”, 2 “some of the time”, 3 “often”, and 4 “most of the time or always”. Appetite was dichotomized as follows: no problems with appetite (answer 1) and poor appetite (answers 2–4) (26).

### Assessment of covariates

Information on socio-demographics (age, sex and education), lifestyle factors (alcohol use and smoking status), co-morbidity (number of chronic diseases, medication use and depression), and cognitive status were all collected by questionnaires.

Education was defined as high (higher vocational, college or university education), medium (general secondary education, lower and intermediate vocational education, and general intermediate education) or low (elementary education completed or not completed). The Garretsen alcohol consumption index was used to categorize alcohol consumption into four categories based on number of times drinking per month and number of drinks consumed each time: excessive, moderate, light, and no alcohol use (27). Smoking status was dichotomized into non-smokers (never or former smoker) and current smoker. For current smokers, the number of cigarettes per week was documented. Three categories were created the number of chronic diseases: no chronic diseases, one chronic disease, and two or more chronic diseases. Medication use was assessed by asking the respondents to show their prescribed medication containers to the interviewers. It was classified into three categories: no medication use, use of one to four medications, and use of five or more medications. Depressive symptoms were dichotomized using the 20-item CES-D scale. A CES-D score above 15 out of 60 indicated depression (25). Cognitive status was measured by the Mini Mental State Exam (MMSE) with a score ranging from 0 to 30. A score of 23 or less was indicative of impaired cognitive function (28).

### Statistical analysis

Participant characteristics are depicted by means ± standard deviations (SD) or medians and interquartile ranges (IQR) for continuous variables. Categorical characteristics are depicted by numbers and percentages (%). Differences among the olfactory categories (normosmic, microsmic and anosmic) were tested using ANOVA or Kruskal-Wallis test (continuous variables) and Fisher's Exact test (categorical variables).

For the main analyses, the UPSIT-score was used as continuous independent variable in regression models. Linear regression analyses were performed to analyze the associations of UPSIT-score with BMI and of UPSIT-score with prospective weight change. First, crude models were made, which were checked for potential effect modifiers: sex, age, smoking status, and appetite. In case of significant effect modification (p<0.10), the data were stratified accordingly. Each model was then adjusted for potential confounders including: education, smoking, alcohol use, number of medications and chronic diseases, MMSE-score, and CESD-score.

The association between olfactory function and appetite was examined with logistic regression. Effect modification and confounding were examined as described above. In order to use CES-D score as covariate in the logistic regression, the appetite question, which was also the outcome measurement, was excluded from calculating the CES-D score.

A two-tailed p value of <0.05 was considered statistically significant. All statistical analyses were performed using SPSS software version 22 (SPSS Inc., Chicago Illinois).

## Results

### *Participants' characteristics*

**Figure 1:**
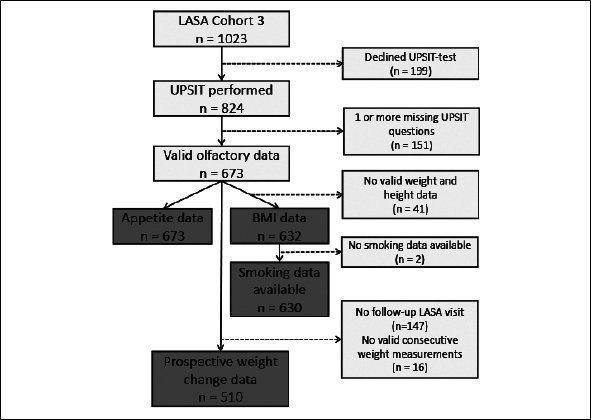
Flowchart of number of participants of the Longitudinal Aging Study Amsterdam (LASA) included in the final analyses LASA: Longitudinal Aging Study Amsterdam; UPSIT: University of Pennsylvania Smell Identification Test; BMI: Body Mass Index.

Of the 824 LASA-participants who had consented to the UPSIT-test, 673 completed all UPSIT-questions and were included in the analyses. Figure 1 depicts the flowchart of all participants.

**Figure 2:**
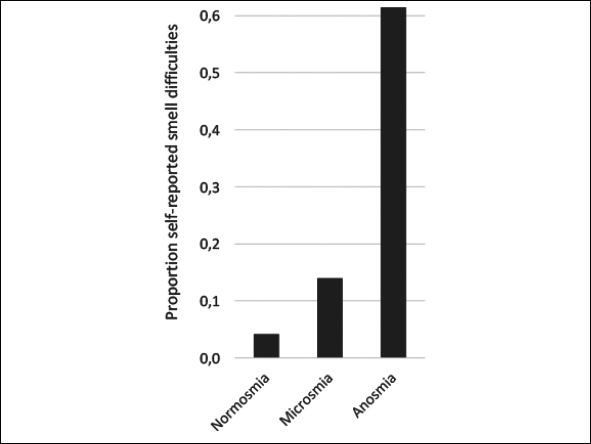
Self-reported smell difficulties across the three olfactory categories based on the University of Pennsylvania Smell Identification Test (UPSIT)

The participant characteristics are shown in table 1. The median (IQR) of the UPSIT-score was 33 (30, 31, 32, 33, 34, 35). Based on their UPSIT-scores, 15 (2.2%) participants were anosmic, 415 (61.7%) were microsmic and 243 (36.1%) were normosmic. The prevalence of self-reported smell difficulties was associated with the olfactory categories. However, about one-third (38.5%) of the participants with functional anosmia did not report having any smelling problems (figure 2).

**Table 1:** Characteristics of the Longitudinal Aging Study Amsterdam (LASA) participants, stratified by olfactory function category based on the University of Pennsylvania Smell Identification Test (UPSIT)

**Characteristics**	**All (n=673)**	**Normosmic (n = 243)**	**Microsmic (n = 415)**	**Anosmic (n = 15)**	P-value*
Age (years)	60.4 [57.8–63.1]	60.4 [57.7–63.1]	60.3 [57.8–63.1]	60.9 [58.2–63.9]	0,79
Male	328 (48.7)	122 (50.2)	201 (48.4)	5 (33.3)	0,451
BMI (kg/m2) (n=632)	27.0 ± 4.6	26.9 ± 5.0	27.2 ± 4.4	25.2 ± 1.8	0,228
Poor appetite	69 (10.3)	23 (9.5)	46 (11.1)	0 (0.0)	0,465
3-year Weight change (kg) (n=510)	−0.66 ± 4.8	−1.1 ± 4.8	−0.5 ± 4.7	1.6 ± 5.0	0,102
Follow-up time (days) (n=510)	1134.6 ± 59.4	1139.4 ± 62.3	1131.6 ± 57.2	1128.8 ± 66.7	0,345
UPSIT-score	33 [30–35]	36 [35–37]	31 [29–33]	16 [12–17]	<0.001‡
Self-reported smell problems (n=592)	68 (11.5)	9 (4.2)	51 (14.0)	8 (61.5)	<0.001‡
Current smoker (n=641)	97 (15.1)	20 (8.4)	75 (19.3)	2 (13.3)	0.001‡
Number of cigarettes/week (n=84)	75.0 [29.8–109.3]	56.0 [19.5–101.3]	84.0 [35.0–118.5]	56	0,185
Alcohol use (n=642)					
*No alcohol use*	80 (12.5)	25 (10.5)	52 (13.3)	3 (20.0)	0,129
*Light*	262 (40.8)	96 (40.5)	157 (40.3)	9 (60.0)	
*Moderate*	249 (38.8)	102 (43.0)	145 (37.2)	2 (13.3)	
*(Very) Excessive*	51 (7.9)	14 (5.9)	36 (9.2)	1 (6.7)	
Number of chronic diseases					
*No chronic diseases*	285 (42.3)	106 (43.6)	172 (41.4)	7 (46.7)	0,939
*1 chronic disease*	260 (38.6)	94 (38.7)	160 (38.6)	6 (40.0)	
*2 or more chronic diseases*	128 (19.0)	43 (17.7)	83 (20.0)	2 (13.3)	
Number of medications (n=641)					
*No medication use*	242 (37.8)	96 (39.5)	138 (40.5)	8 (53.3)	0,319
*Use of 1 to 4 medications*	316 (49.3)	116 (47.7)	195 (48.9)	5 (33.3)	
*Use of 5 or more medications*	83 (12.9)	25 (10.3)	56 (17.7)	2 (13.3)	
Poor cognitive status (MMSE ≤; 23)	11 (1.6)	4 (1.6)	6 (1.4)	1 (6.7)	0,307
Depressive symptoms (CESD ≥ 16) (n=671)	80 (11.9)	23 (9.5)	55 (13.3)	2 (13.3)	0,292
Education					
*Low*	52 (7.7)	13 (5.3)	38 (9.2)	1 (6.7)	0.001‡
*Medium*	402 (59.7)	128 (52.7)	262 (63.1)	12 (80.0)	
*High*	219 (32.5)	102 (42.0)	115 (27.7)	2 (13.3)	


Data is depicted in mean ± SD, median [IQR] and number (%); SD: Standard deviation; IQR: Interquartile range; UPSIT: University of Pennsylvania Smell Identification Test; BMI: body mass index; CESD: Center of Epidemiologic Studies Depression scale; MMSE: Mini-Mental State Exam; *Normally distributed continuous data analyzed with ANOVA-test; non-normally distributed continuous data analyzed with Kruskal-Wallis test; categorical data analyzed with Fisher's exact test; ‡P-value below 0.05.

In our sample, subjects with microsmia and anosmia had a lower level of education: 72% of the microsmic group and 86.7% of the anosmic group had low or medium educational levels compared to 58.0% of the normosmic group. Also, participants with microsmia and anosmia were more likely to be current smokers (19.3% and 13.3%, respectively) than participants with normosmia (8.4%). There was no difference in the number of cigarettes smoked per week among the smokers in the olfactory categories. For the baseline body weight, self-reported instead of measured weight was used in 5 cases (0.8%). No self-reported weight needed to be used for follow-up measurements. Univariate comparison of BMI, appetite and weight change did not reveal significant differences among the three olfactory categories.

### Olfactory function and BMI

The crude linear regression analysis revealed no significant association between UPSIT-score and BMI. Smoking status was found to be the only statistically significant effect modifier (p=0.009). In the smokers, lower UPSIT-score — indicating poorer olfactory function — was associated with lower BMI (B=0.225, SE=0.078, p=0.005, table 2). No such association was observed among non-smokers (B=−0.042, SE=0.045, p=0.341). The association of olfactory function with BMI in smokers remained statistically significant after adjusting for confounders (B=0.178, SE=0.081, p=0.032).

**Table 2:** The crude and adjusted associations of olfactory function with BMI, poor appetite and 3-year weight change in older participants of the Longitudinal Aging Study Amsterdam

	**B**	**OR 95%-CI**	**P-value**
**Association olfactory function — BMI**			
**Smokers (n=96)**			
Crude model no. 1	0,225	0.071–0.379	0.005*
Adjusted model no. 2^a^	0,178	0.016–0.340	0.032*
**Association olfactory function — BMI**			
**Non-smokers (n=534)**			
Crude model no. 1	−0,042	−0,175	0,341
Adjusted model no. 2^a^	−0,015	−0,172	0,732
**Association olfactory function-poor appetite**			
**All participants (n=673)**			
Crude model no. 1	1,029	0.971–1.090	0,337
Adjusted model no. 2^b^	1,062	0.981–1.150	0,137
**Association olfactory function — prospective 3-year weight change**			
**All participants (n=510)**			
Crude model no. 1^c^	−0,033		0,447
Adjusted model no. 2^d^	−0,027		0,548


Shown are regression coefficients (B), Odd ratio (OR), 95% confidence interval (95%-CI), and P-values; *P-value below 0.05; a. adjusted for age, sex, alcohol use, education, chronic diseases, medication use, MMSE, CES-D; b. adjusted for age, sex, smoking status, alcohol use, education, chronic diseases, medication use, MMSE, CES-D (appetite question excluded) and BMI; c. adjusted for baseline weight and follow-up time in days; d. adjusted for baseline weight, follow-up time in days, age, sex, smoking status, alcohol use, education, chronic diseases, medication use, MMSE, CES-D.

### Olfactory function and appetite

Using binary logistic regression, no association was observed of UPSIT-score with poor appetite in the crude model (OR=1.029, p=0.337), or the adjusted model (OR=1.062, p=0.137). No effect modification was found.

### Olfactory function and prospective weight change

Linear regression revealed no association of UPSIT-score with 3-year weight change in the crude model (B=−0.033, SE=0.044, p=0.447) or the adjusted model (B=−0.027, SE=0.045, p=0.548). Both models were additionally adjusted for baseline weight and follow-up time in days. No significant effect modification by sex, age, smoking status, or appetite was found.

## Discussion

Lower olfactory function scores were associated with lower BMI in older adults who smoke, but not in older adults who do not smoke. While the negative influence of smoking itself on olfactory function has long been established, the effect-modifying role of smoking has not been reported before.

There are several possible explanations as to why poor olfactory function might be differently associated with BMI in smokers and non-smokers. Any association of olfactory function with BMI may normally be overshadowed by other factors that compensate for the loss of smell in older adults. For instance, an increased preference for specific food texture (like creamy foods) or taste (like salty and sugary foods) has been reported (15), and was suggested to secure adequate caloric intake in older adults with impaired olfactory function (14, 15, 18). Possibly these dietary adaptations to the loss of smell differ for smokers and non-smokers. Since we did not measure food intake or dietary choices, we cannot say whether this was the case in our cohort. Furthermore, central appetite regulation (e.g. by the secretion of hunger-hormone ghrelin and orexogenic neurons in the hypothalamus) is likely to safeguard adequate food intake, even in the absence of smell (29). Nicotine, however, has been demonstrated to modulate the effects of ghrelin and promote anorexogenic neuron activation in the hypothalamus (30). With the modulation of central appetite regulation by nicotine, the relative influence of olfactory function on appetite and food intake could become more prominent in smokers than non-smokers. Additionally, our smokers had lower median [IQR] UPSIT-scores than our non-smokers (31 (28, 29, 30, 31, 32, 33, 34) versus 33 (31, 32, 33, 34, 35), respectively (p-value < 0.001)). The more severe olfactory dysfunction in smokers is likely to affect nutritional status to a greater extent than the milder olfactory dysfunction in non-smokers.

To evaluate the association between smoking and olfactory function further, we performed a post-hoc analysis in which we tested the differences in continuous UPSIT-score among current-, former-, and never smokers, using a Kruskall-Wallis test with Bonferroni correction. There was a significant difference in median UPSIT-score between never smokers and current smokers (33 versus 31, respectively, adjusted p=0.002) and between former smokers and current smokers (33 versus 31, respectively, adjusted p=0.001), but not between former smokers and never smokers (33 versus 33, adjusted p=1.000). This is in line with the results of Murphy et al. (8), suggesting that the impact of smoking on olfactory function is reversible. We did not find the earlier reported dose-dependent relationship among smokers (31) as the number of cigarettes smoked per week did not differ among the olfactory function categories.

Our results show that olfactory function is not significantly associated with appetite. This is in line with Arganini et al. (17), but contrasts the findings of de Jong et al. (16). It is noteworthy that only the latter study assessed appetite by multiple questions instead of one. We cannot exclude that a single question may lack sensitivity in detecting poor appetite. However, poor appetite using this single question has been shown to be predictive of future incidence of malnutrition (1).

Our study is the first to investigate the association of olfactory function with prospective weight change. However, we did not find the two to be significantly associated. This might be due to the singular measurement of olfactory function. It is not known for how long any olfactory impairments existed and to what extent this had already influenced nutritional status. We cannot preclude that the onset of olfactory impairments was still recent in the relatively younger and more vital older adults in our cohort. Olfactory impairments may therefore not yet have impacted nutritional status (32). Contrarily, if olfactory impairments were more long-standing, they could have already caused food-intake and energy balance to shift towards a new equilibrium in which the participants' weight are stable.

The association we and others (11, 33) found between education and olfactory function scores could be explained by an advantage of higher educated participants in correctly carrying out a smell identification test. Consistent with literature (8, 11, 17, 18, 20), self-awareness of impaired olfactory function in our study population was low with 38.5% of anosmic participants reporting no smell difficulties. Possibly, the underreporting 38.5% represent the proportion of participants who have been anosmic for a long period of time. They might therefore not have recognized a decline in olfactory function. Still, these findings demonstrate the need for objective olfactory tests when olfactory dysfunction is suspected. There was also no difference in gender among olfactory function categories, which can be contributed to the gender-specific cutoffs for these groups. When using the Mann-Whitney U test to assess the difference in continuous UPSIT-score between men and women, women had higher UPSIT scores (p=0.013), consistent with literature (14, 20, 34, 35).

There are several strengths to the present study. First, the study includes a large representative sample. Additionally, because LASA included data on many life domains, there was ample opportunity to correct for potential confounders. However, residual confounding can never be excluded. Third, olfactory function was objectively measured by a valid, extensive 40-item standardized test with high test-retest reliability (r=0.918)(36).

A first limitation of the study is the potential selection bias. Out of 1023 LASA-participants, 199 declined taking the UPSIT-test. Of those taking the test, 151 had one or more missing items in the UPSIT-test and were excluded from the analyses. Testing the differences in characteristics between the included and excluded participants revealed that participants who declined taking the UPSIT-test or had incomplete tests were more likely to be smokers than those who had completed the UPSIT-test (29.5% and 22.0% versus 15.1%, respectively). They were also more likely to have a poor cognitive status (7.0% and 5.3% versus 1.6%, respectively) and a lower education level (12.1% and 19.2% versus 7.7%, respectively). Both smoking and low education were associated with lower UPSIT-scores in our analytic sample. To investigate the potential impact of excluding those with incomplete tests, we performed multiple imputation on the missing UPSIT-items and repeated the regression analysis in a total of 767 older adults on UPSIT-score and BMI. The findings were similar to the current results. Again there was effect modification by smoking status (p=0.030), and again both the crude and adjusted model showed a significant positive association of UPSIT-score with BMI in smokers (adjusted regression model B=0.175, SE=0.075, p=0.020), but not in non-smokers (adjusted regression model B=0.022, SE=0.039, p=0.547). These results suggest that potential selection bias did not affect the conclusion of our study.

A second limitation is that we only used an odor identification test and not an additional threshold test. It is possible that perceiving a scent rather than correctly identifying it, is more important in making food related choices. Therefore, using an identification test may have caused an underestimation of any association of olfactory function with nutritional status. It must be noted, however, that threshold tests are time-consuming and might be considered too tiresome (19). Furthermore, typically only one non-food odor is tested in threshold tests, which decreases its validity to objectify overall olfactory function in relation to nutrition.

A final limitation is the cross-sectional design of our study. Hence, we cannot assume a cause-effect relationship between olfactory function and BMI in smokers. Future prospective studies using repeated measurements of both olfactory function, dietary intake and nutritional status in the young-old and old-old are therefore needed.

In conclusion, lower olfactory function scores were associated with lower BMI in smokers, but not in non-smokers. Smoking older adults with poor olfactory function might therefore pose as a vulnerable group for the development of undernutrition. Lower olfactory scores were not associated with poorer appetite or prospective weight loss in our sample of community-dwelling Dutch older adults, aged 55–65 years. More prospective studies are needed in which different aspects of olfactory function are determined, including identification and threshold tests. Moreover, more data on nutritional status, such as body composition, dietary patterns, and food preferences need to be collected to elucidate the precise nature of the relationship between olfactory function and nutritional state in older persons.
